# Motor Neuron Syndrome as a New Phenotypic Manifestation of Mutation 9185T>C in Gene MTATP6

**DOI:** 10.1155/2014/701761

**Published:** 2014-12-08

**Authors:** Marisa Brum, Cristina Semedo, Rui Guerreiro, José Pinto Marques

**Affiliations:** ^1^Neurology Department, Centro Hospitalar de Setúbal, Rua Camilo Castelo Branco, 2910-446 Setúbal, Portugal; ^2^Instituto de Medicina Molecular, Faculty of Medicine of Lisbon University, 1649-028 Lisbon, Portugal

## Abstract

*Background.* The mutation 9185T>C in ATP6 gene, associated with Leigh syndrome, was reported in only few families. Motor neuron disease (MND), both clinically and electrophysiologically, was not previously described in association with this mutation. *Case Report.* 33-year-old male, with family history of mitochondrial disease, presented with cognitive impairment, exercise intolerance, and progressive muscle weakness. Examination revealed global hypotonia, and proximal tetraparesis, without atrophy or fasciculation, pyramidal signs, or sensory symptoms. The laboratory findings revealed an increase of lactate and lactate/pyruvate ratio; electromyogram showed chronic neurogenic compromise; muscle biopsy was suggestive of spinal muscular atrophy and mitochondriopathy; genetic study of SMN1 was negative but detected a homoplasmic mutation 9185T>C in ATP6 gene. His younger sister, with the same mutation, had cognitive impairment, ataxia, and muscle weakness. EMG showed axonal peripheral neuropathy. *Conclusion.* This case is unique because of the benignity and the coexistence of clinical, neurophysiological, and pathological findings suggestive of MND that, although described in mitochondrial disease, have not yet been reported in association with 9185T>C mutation. The present case contributes to the expansion of the phenotypic expressions of this particular mutation.

## 1. Background

The mitochondriopathy resulting from the mutation in gene MTATP6 has an extremely variable clinical phenotype including Leigh syndrome (LS), neurogenic muscle weakness, ataxia, retinitis pigmentosa (NARP), and occasionally asymptomatic patients [1]. The most frequent clinical presentation is an infantile-onset Leigh syndrome [1]. LS typically begins before 6 months of age with brainstem dysfunction, progressive psychomotor decline, and death within 2 years [2]. Moreover, it is characterized by optic atrophy, ocular movement abnormalities, ptosis, respiratory disturbances (due to brainstem dysfunction), pyramidal signs, and less frequently dystonia, ataxia, peripheral neuropathy, deafness, and seizures [3, 4]. In addition, cardiomyopathy, liver, kidney, and gastrointestinal disorders may be part of the clinical picture [4]. Diagnosis rests on clinical features combined with high levels of lactate and pyruvate in plasma, cerebrospinal fluid, or both; neuropathological findings, characteristic signal abnormalities in the basal ganglia and brainstem on MR imaging, and finally mitochondrial gene analysis [5]. There are many mutations related to Leigh syndrome; however, to our knowledge, the specific mutation, 9185T>C in ATP6 gene, was only described in 5 families [2, 3, 6–8], featuring varied clinical manifestations.

Mitochondriopathy has been reported rarely to mimic motor neuron disease, in particular, spinal muscular atrophy (SMA) [9, 10] and amyotrophic lateral sclerosis [11]. SMA is an autosomal recessive disorder characterized by degeneration of the anterior horn cells of spinal cord, resulting in progressive paralysis with muscular atrophy. The diagnosis is based on symptoms of muscle weakness and signs of denervation on electrophysiologic studies and muscle histopathology. The deletion analysis of the survival motor neuron (SMN) gene is useful to confirm the diagnosis [10].

The authors describe two siblings of a family with the rare mutation 9185T>C in ATP6 gene, one with phenotype suggestive of motor neuron syndrome, not previously described.

## 2. Case Report

**Figure 1:**
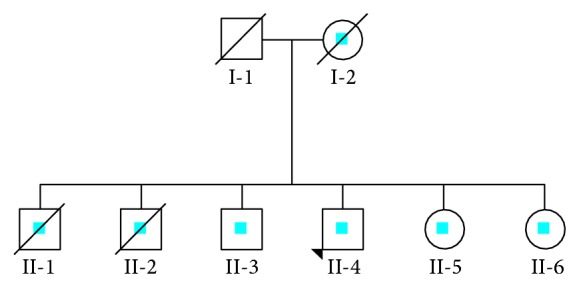
Pedigree of family with mitochondriopathy. I-1: death by suicide at 61; I-2: death from haemorragic stroke at 48; II-1: Leigh syndrome, death by suicide at 16 A; II-2: Leigh syndrome, death at 34 A, from respiratory failure; II-3: 34 A, epilepsy, cognitive impairment, and polyneuropathy; II-4: 33 A, index case; II-5: 17 A, cognitive impairment, ataxia, and peripheral neuropathy; II-6: 13 A, neurodevelopmental delay.

This included 33-year-old, Caucasian male, 75 kg in weight and 1.66 meters in height, born through a normal pregnancy and delivery. Fourth sibling of nonconsanguineous parents, with family history of mitochondrial encephalopathy (Figure 1), his mother with congenital deafness, died at age 48 due to an haemorrhagic stroke; two older brothers died at ages 16 and 34, with the diagnosis of Leigh syndrome; the patient's 34-year-old brother revealed cognitive impairment, polyneuropathy, and epilepsy; and two younger sisters revealed cognitive impairment, ataxia, peripheral neuropathy, and neurodevelopmental delay, respectively. The patient presented with fatigue, exercise intolerance, and learning difficulties since childhood. Despite cognitive deficits the patient lived independently with full function of daily living skills. In the last year he denoted progressive decline of the muscle strength. At neurological examination overall hypotonia and proximal tetraparesis, normal deep tendon reflexes in upper and lower extremities, and plantar flexor response bilaterally were present. We did not observe other signs, like atrophy, pyramidal signs, sensory, cerebellum, or cranial nerves dysfunction. For the remainder of physical examination, the patient had abnormalities of the joints, including hammertoes. There were no other systemic changes, including cardiac, endocrine, and hepatic dysfunction and no history of seizures.

**Figure 2:**
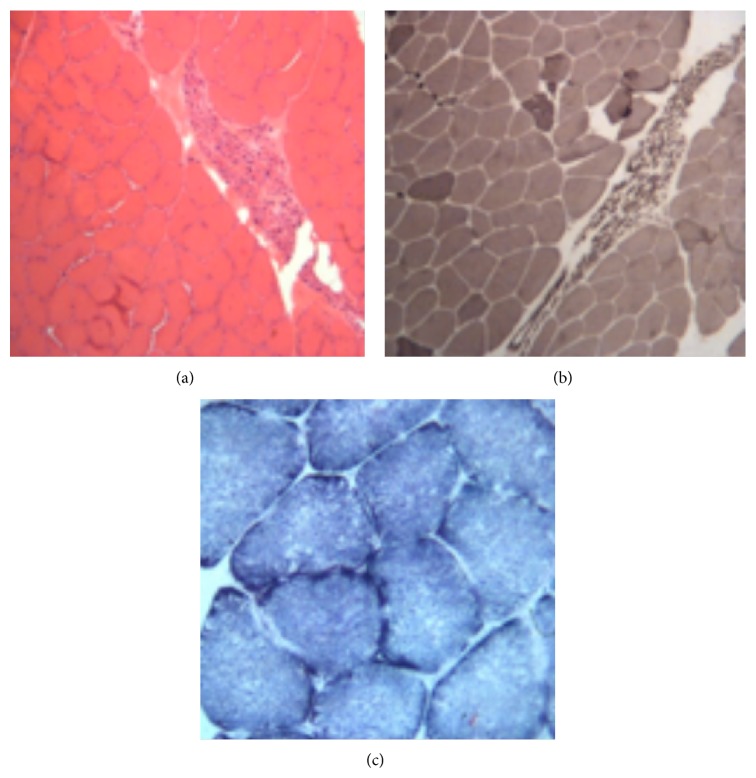
Histopathologic finding on muscle biopsy. (a) H&E: large group atrophy (atrophic muscle, an issue surrounded by other normal appearances of fascicles or scattered angular atrophic fibers). (b) ATPase reaction aspects of type-grouping, typical neurogenic muscular atrophy; muscular fascicle and the atrophic consisting of a single type of fiber; in this case type I and the other parts are nearly all composed of fibers of type II. (c) Oxidative reaction SDH aggregates subsarcolemmal hyperchromatic, crescent-shaped, which correspond to the accumulation of mitochondria.

Routine laboratory analyses like haemoglobin, haematocrit, white cell count, differential white cell count, platelet count, serum ion levels, albumin, glucose, creatine kinase activity, ammonia, and liver and kidney functions were normal; spinal cord and brain MRI were normal, with no significant foci of abnormal signal in the brain or in the cerebellum parenchyma; ECG and transthoracic echocardiography were normal. The remaining study highlights an increase in the determination of serum lactate 2.95 mmol/L at rest and 2.78 mmol/L after exercise (reference values 0.95–2.30 and 0.80–1.50, resp.) and lactate/pyruvate ratio of 25.0 at rest and 20.9 after exercise (ratio normal 10–28 and 11–18, resp.), as well as elevated cerebrospinal fluid lactate; the nerve conduction studies (NCS) showed normal sensory studies but CMAP studies of upper and lower limbs were slightly reduced with normal range latency and velocity and without features of conduction block. The electromyogram (EMG) showed no spontaneous activity, polyphasic motor unit potentials (MUPs) with increased amplitude, increased duration, and decrease recruitment on right* first dorsal interosseous muscle*,* extensor digitorum communis*,* biceps brachialis*,* tibialis anterior*,* medial gastrocnemius*, and* vastus lateralis* in lower limbs. Repetitive stimulation did not reveal decrement. NCS and EMG revealed signs of chronic neurogenic compromise that could suggest motor neuron dysfunction. Biopsy of muscle and nerve exhibited marked variation in the fiber size by the presence of large groups of muscle atrophy and hypertrophy of muscle fibers on healthy fascicles, “fiber-type-grouping;” a slight increase in the number of nuclear centralizations, lipid accumulation, and crescent-shaped subsarcolemmal hyperchromatic aggregates on oxidative reaction SDH corresponded to the accumulation of mitochondria. We were not able to detect clear “ragged-red fibers” in Gomori Trichrome stain. These features are suggestive of spinal muscular atrophy (SMA) with some mitochondrial alterations (Figures 2(a)–2(c)). EEG showed generalized slowing without epileptiform discharges, with no ophthalmologic signs such as retinitis pigmentosa or optic atrophy. A genetic study revealed an apparent homoplasmic T>C substitution at nucleotide position 9185, in MTATP6, that replaces a highly conserved leucine to proline at codon 220 (p.L220P); the genetic study of SMA did not detect any homozygous deletion in exon 7 or heterozygosity of exons 7 and 8, in the SMN1 gene. The mitochondriopathy was confirmed, according to clinical and genetic criteria of* Nijmegen Clinical Criteria for Mitochondrial Disease* [12] despite the atypical features. The patient performed thiamine (50 mg/day), coenzyme Q10 (400 mg/day), and L-carnitine (4000 mg/dia), for 1 year, with slight clinical improvement.

The patient's sister, 22-year-old fifth sibling, revealed mild cognitive impairment, ataxia, and exercise intolerance 6 years ago. Like her brother she was independent of activities of daily living. At exam we observed hypertelorism,* pes cavus* and hammertoes, proximal muscular weakness, and ataxia. The laboratory study highlights an increase of lactate and lactate/pyruvate ratio in serum, at rest and after exercise; EMG revealed axonal peripheral neuropathy, predominantly in lower limbs; MRI showed cerebellar atrophy and muscle biopsy were normal. Cardiac study with ECG and transthoracic echocardiography were normal. The genetic study displayed homoplasmic m.9185T>C mutation. The remaining study was normal.

## 3. Conclusion

This specific mutation, 9185T>C in ATP6 gene, was described in only 5 families, associated with Leigh syndrome and characterized by phenotypic variability. Ataxia and peripheral neuropathy were the prominent features in the reports of a Canadian family with late-onset LS [3]. Childs et al. [2] related variability in the phenotypes of the same family, including late-onset LS, NARP, and isolated demyelinating peripheral neuropathy. Another case report referred to a 3-year-old child presenting a clinical picture of a fulminate onset ataxia, exacerbated muscle fatigue, bilateral ptosis, dysarthric speech, and abnormal breathing that required a mechanical ventilation; in this case, disease exacerbation was related to febrile viral-like illness; MRI showed hyperintensity in the periaqueductal region and bilateral basal ganglia on T2 and FLAIR [6]. Furthermore, Moslemi and colleagues [7] described a 7-year-old child who suddenly developed bilateral ptosis, ophthalmoparesis, and generalized fatigue; MRI of the brain showed increased signal periaqueductally in the mesencephalon and in the dorsal part of the brainstem; the clinical picture was reversible with the oral treatment. Different from previous descriptions, Pfeffer et al. [1] related five siblings in the same generation presented with adult-onset unexplained ataxia, clinically indistinguishable from other spinocerebellar ataxia syndromes. After excluding common sporadic and inherited causes, they found m.9185T>C in all family members. More recently, Auré and colleagues [8] described acute episodes of limb weakness mimicking periodic paralysis due to channelopathies, in a family with homoplasmic m.9185T>C p.Leu220Pro.

Previous studies have showed that mitochondriopathy occasionally mimics motor neuron disease, phenotypically and electrophysiologically. Pons et al. [10] reported a case with mitochondrial myopathy simulating SMA without deletion in the SMN gene, but with decreased COX and succinate-cytochrome c reductase activities in combination with mitochondrial DNA depletion. Rubio-Gozalbo et al. [9] reported a child with an SMA-like picture, cardiomyopathy, neurogenic EMG, COX negative muscle biopsy, and increased serum and cerebrospinal fluid lactate. No SMN gene mutations were found. Also, Finsterer [11] reported women with alleged family history of ALS, clinical and electrophysiological features of motor neuron disease, negative superoxide-dismutase gene mutation, and muscle histopathology suggestive of mitochondriopathy. The mtDNA investigation revealed substitution in the isoleucine tRNA in ATPase-6.

The present mutation, 9185T>C in MTATP6, with clinical, electrophysiologic, and histopathologic findings resembling SMA, has never been reported. The present case shows a male patient with documented mutation in mitochondrial DNA, family history of Leigh syndrome, a clinical picture, EMG, and histopathology suggestive of a motor neuron disease. The present family had phenotypic variability, but unlike previously described in literature these two siblings had an indolent clinical picture, unrelated to febrile syndromes and nondisabling presentation. Another unusual feature in these cases is the absence of multisystem involvement, frequent in Leigh syndrome.

The mitochondriopathy with the mutation 9185T>C in gene MTATP6 of mitochondrial DNA is rare. The present case distinguishes itself even more by the relative benignity and the coexistence of clinical neurophysiological and pathological findings suggestive of motor neuron disease. Although described in mitochondrial disease, such clinical spectrum has not yet been reported in association with this particular mutation. Motor neuron disease features with cognitive impairment suggest mitochondrial disorder. The authors propose that in patients with SMA clinical features with the absence of SMN gene mutation, search for mitochondrial mutation, principally those that have family history.
